# The Diversity of Forensic Entomofauna Associated With Decaying Chicken Liver in the Tropical Region of Kerala, India

**DOI:** 10.7759/cureus.50991

**Published:** 2023-12-23

**Authors:** Bincy Benny, Jasimudeen Sulaiman

**Affiliations:** 1 Department of Forensic Medicine, Madurai Kamaraj University, Madurai, IND; 2 Department of Research, National Institute of Physical Medicine and Rehabilitation, Thrissur, IND; 3 Department of Library, St. Stephen's College, Uzhavoor, Uzhavoor, IND

**Keywords:** postmortem interval estimation, insect evidence, arthropod communities, forensic entomology, insect succession

## Abstract

Forensic entomology

To elucidate the time of death based on insect evidence, there are several studies on forensic entomology on life cycles, environmental factors, and feeding habitats of insects. However, there have not been any comprehensive studies on forensic entomology and its usage in forensic inquiry specific to the region, especially Kerala, India. The insect succession on decomposed animal matter plays an important role in estimating minimum postmortem intervals (mPMI).

Objective

The purpose of the study was to understand forensically important insect groups and their role in the decomposition process of dead decaying matter. The different decomposition stages of a corpse vary in attraction to necrophagous insects and the insect fauna depending on its prevailing conditions of decay. The decomposition is highly dependent on the exposition of animal matter and abiotic and biotic factors acting on it. The main objective of the present investigation was to identify the insect fauna associated with decaying chicken liver. The study also envisages comprising the diversity and abundance of insects between two different treatments of animal matter: in contact with soil and controlled conditions in a clean basin.

Method

The study was conducted for 45 days (until the total decomposition of the samples in both conditions) during the pre-monsoon months of April to May 2022 at Chalikadavu, Muvattupuzha, in the Ernakulam district, Kerala, India. The samples were however kept away from direct sunlight and rain to avoid the direct impact on the orienting fauna. The entomofauna found to be associated with the decaying animal matter was carefully collected from the site and stored in 70% isopropyl alcohol for preservation. The total number of insects was recorded along with the hours of maximum incidence, and samples were stored in plastic vials for further identification.

Result

In this study, we analyzed the activities of ants, mites, wasps, cockroaches, moths, beetles, and flies during the decomposition of decaying chicken liver. Among these insects, flies and beetles are two important arthropod communities associated with animal matter decomposition. We collected these foraging organisms for morpho-taxonomic identification. The decomposition stages among the two treatments could help to understand the variable factors in the decomposition of decaying corpse with special reference to the insect fauna acting on it.

Conclusion

We got 100 specimens comprising 28 species in 17 families from Blattidae, Coleoptera, Diptera, Hymenoptera, and Lepidoptera. Besides this, we identified two parasitic wasps with their host (dipteran pupa), which is helpful in postmortem interval (PMI) estimation. Our analysis showed an association between decay and the activity of carrion insects. The decomposition stages among the two treatments could help to understand the variable factors in the decomposition of a decaying corpse with special reference to the insect fauna acting upon it.

## Introduction

The scope of studies on forensic entomology is the use of information obtained from the entomological fauna to investigate an incident of forensic importance, mostly related to the death of an individual. In such cases, forensic entomology uses insects and other arthropods as evidence in legal investigations. Arthropods are mainly employed in homicide, suicide, and mysterious death cases, because insect evidence can provide some circumstances of death [1,2]. When a person's body or an animal begins to decompose immediately after death, a wide array of attributes initiate the breakdown process [3]. During an animal's body transformation from a corpse to skeletal remains, various physical and chemical changes occur on the carcass; these processes are continuous. Several scientists identified eight distinct phases of decomposition [4]; others identified six stages while studying pig carcasses: fresh, bloated, active decay, advanced decay, dried, and remnants [5]. Later, the decomposition stages were reduced to four stages: fresh, bloat, decay, and dry [6]. The above description of the various phases of decomposition is based on appearance and is more subjective in approach; the description of each stage lacks a legible beginning and defined end, especially the dry stage [6]. However, five decompositional phases (fresh, bloat, active decay, advanced decay, and dry/skeletal) have come into use in forensic studies [7].

In cases of untimely death or murder, the postmortem interval (PMI), the calculation of time since death, usually plays a significant role in the investigation, and it is a common task of forensic entomologists [8]. They investigate the damage that the organisms have caused to the cadaver and other arthropods connected to the cadaver or carrion to determine the postmortem interval [9]. Therefore, forensic entomology deals with the types of insect eggs that appear, where they appear on the body, in what order, etc. Flesh flies are one of the dominant necrophagous insects during the early stages of decomposition, and such forensically important organisms include mites, ants, beetles, birds, and mice [9,10]. Therefore, forensic entomology investigates the invasion of the orientation pattern of insects, including ants, flies, beetles, wasps, moths, and mites, in a rotting corpse [11]. At first, a study on a human carcass from Germany in a suspected homicide case led to the development of an insect collection kit to standardize the procedure; the study was mainly focused on calculating the postmortem interval and used a DNA-based reliable method for insect group identification [12,13]. Later, the role and types of arthropods and other insects in forensic investigations formed a common standard guideline for police officers, pathologists, and entomologists for crime investigations. In cases of suspicious deaths, the minimum time since death can be further determined by estimating the age of the oldest necrophagous insects that developed on the cadaver or by analyzing the arthropod species composition on the cadaver through molecular and taxonomic analysis of corpses [13,14].

Recently, a general overview of insect succession patterns and toxicological studies, along with insect identification using an assortment of animal models and some human case studies, was also referred, which will facilitate the expansion of forensic entomology and open its use in criminal probes [15]. Therefore, many studies have been undertaken in forensic entomology, including the life cycle, environmental factors, and feeding habitats of forensically essential organisms. However, in-depth studies on identifying forensically important wasps and moths are lacking. More importantly, no comprehensive studies on forensic entomology and its usage in forensic inquiry are specific to the region. For the research, as mentioned earlier, we conducted a study on decaying animal matter (chicken liver) to understand the relationship between insects and cadavers in terms of their forensic importance in the region of Kerala, India.

## Materials and methods

Study area

**Figure 1:**
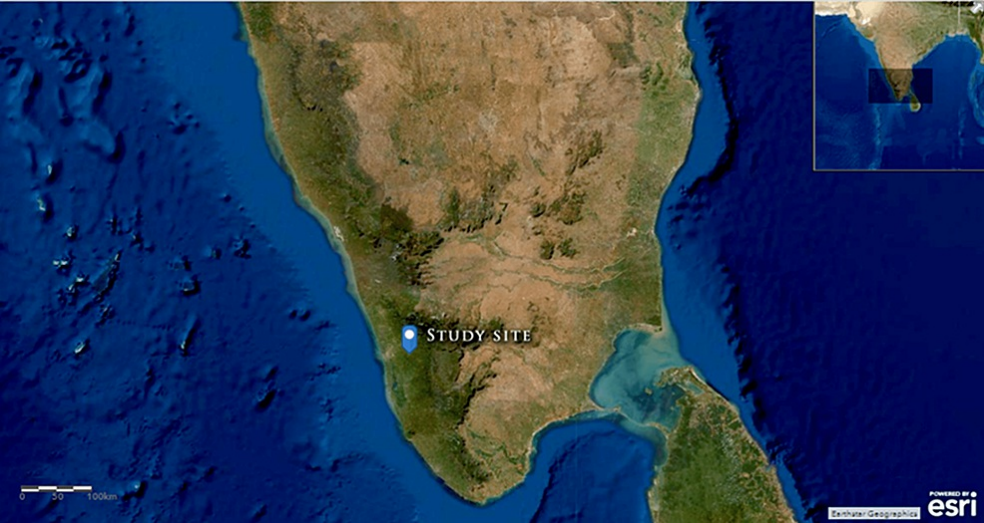
Study Site

We conducted the experiment during the pre-monsoon months of April to May 2022 at Chalikadavu, Muvattupuzha (9°59'20.7'' N, 76°35'21.9'' E), in the Ernakulam district, Kerala, India (Figure 1). The mean average temperature falls at 28°C, with the maximum reaching as high as 34°C during the day and a minimum of 23°C at night, with humidity at 70%-80% (except during intermittent rain). There was no rainfall during the initial days, followed by intermittent showers, which may have influenced the study's outcome. However, we kept samples away from direct sunlight and rain to avoid their direct impact on the orienting fauna.

The animal model

**Figure 2:**
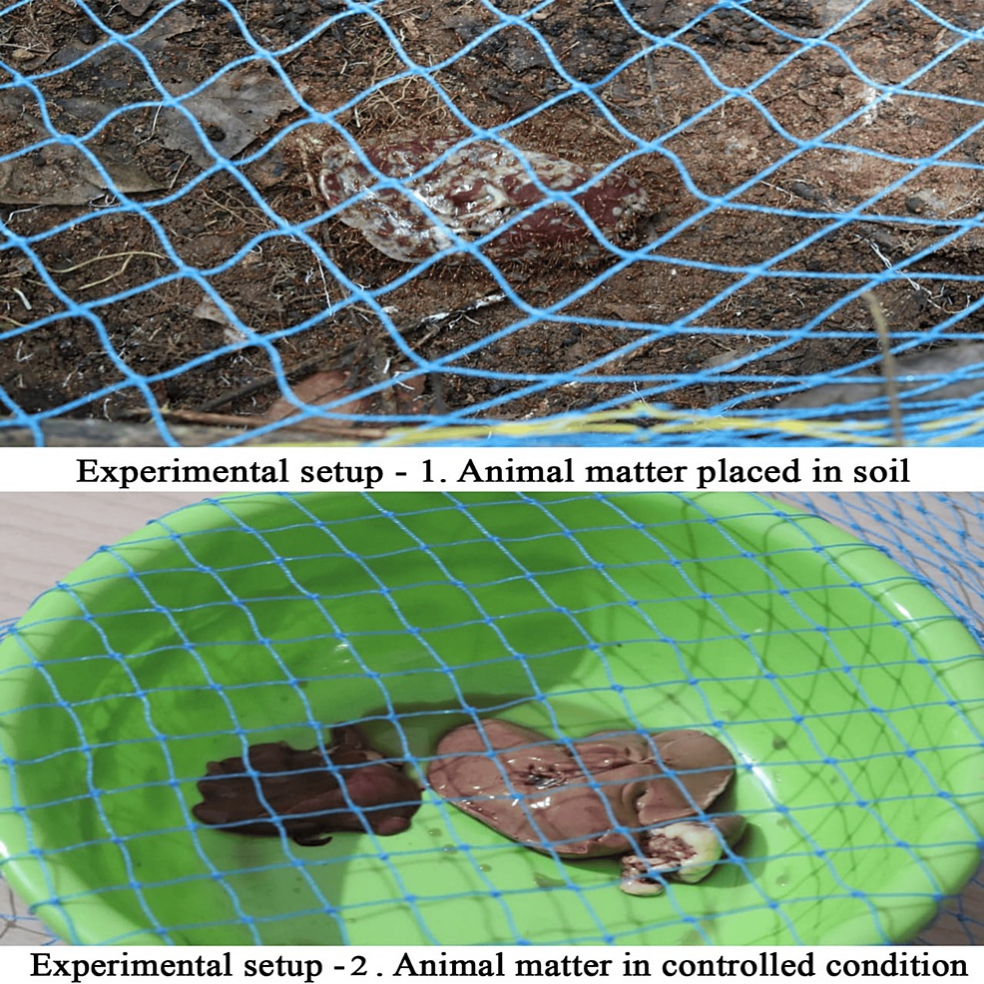
Experimental Setup

We used chicken liver (n = 2, approximately 125 g) as an animal model. The freshly cut and trimmed chicken was procured from a local vendor. We sealed samples separately in double plastic bags and transported and placed them at their respective decomposition sites before 0700 hours on day 1. We put samples in a basin and soil, separated by a minimum distance of 25 m (by using measuring tape) to reduce insect orientation to and from the adjacent batch (Figure 2). To protect the samples from scavenging rodents and larger organisms, we covered each of the samples with a large plastic mesh (50 cm × 50 cm), which was wide enough to facilitate insects' entry into the sample. The dry mass of the substrate was taken for the controlled sample, while it was virtually impossible to ascertain that of the one placed in the soil.

Observations on the entomofauna

We observed the experimental setup at a two-hour interval from 0700 hours to 2200 hours during the first seven days. Later, the frequency of observations made at two-, four-, eight-, 16-, and eight-day intervals was calculated. The observations were made three times a day, from 0800 hours to 2200 hours. Various insects visited the sample in the first hours, and the images were captured using a digital camera. The insects that visited but were not seen feeding or spending more than 2-3 minutes on the substrate were not included in the study because they were not contributing much to the decomposition of the substrate. The entomofauna associated with the decaying animal matter was carefully collected from the site by using an insect sweep net and stored in 70% isopropyl alcohol for preservation. We recorded the total number of insects and the hours of maximum incidence. Then, the collected insect specimens were stored in plastic vials for further identification.

Species identification

We classified the samples into orders based on their visible external appearance. The specimens were pinned using standard entomological pins for taxonomic identification. The collected fauna was stored in 70% isopropyl alcohol. Soft-bodied insects were air-dried and preserved in airtight containers. We used a stereo zoom microscope to identify specimens, and good images were taken using the attached digital camera. For species-level identification, a standard taxonomic key, "Study of Insects" [16], was used. Besides employing the guidance of experts to identify them to the genus and species level, all diagnoses are made using the original descriptions of the taxa with the help of taxonomists. In this study, we discuss a comparative analysis of insects that are specific and frequent visitors to decaying animal matter, with particular emphasis on Diptera and its parasitoids of the Hymenoptera order.

## Results

Decomposition process

**Figure 3:**
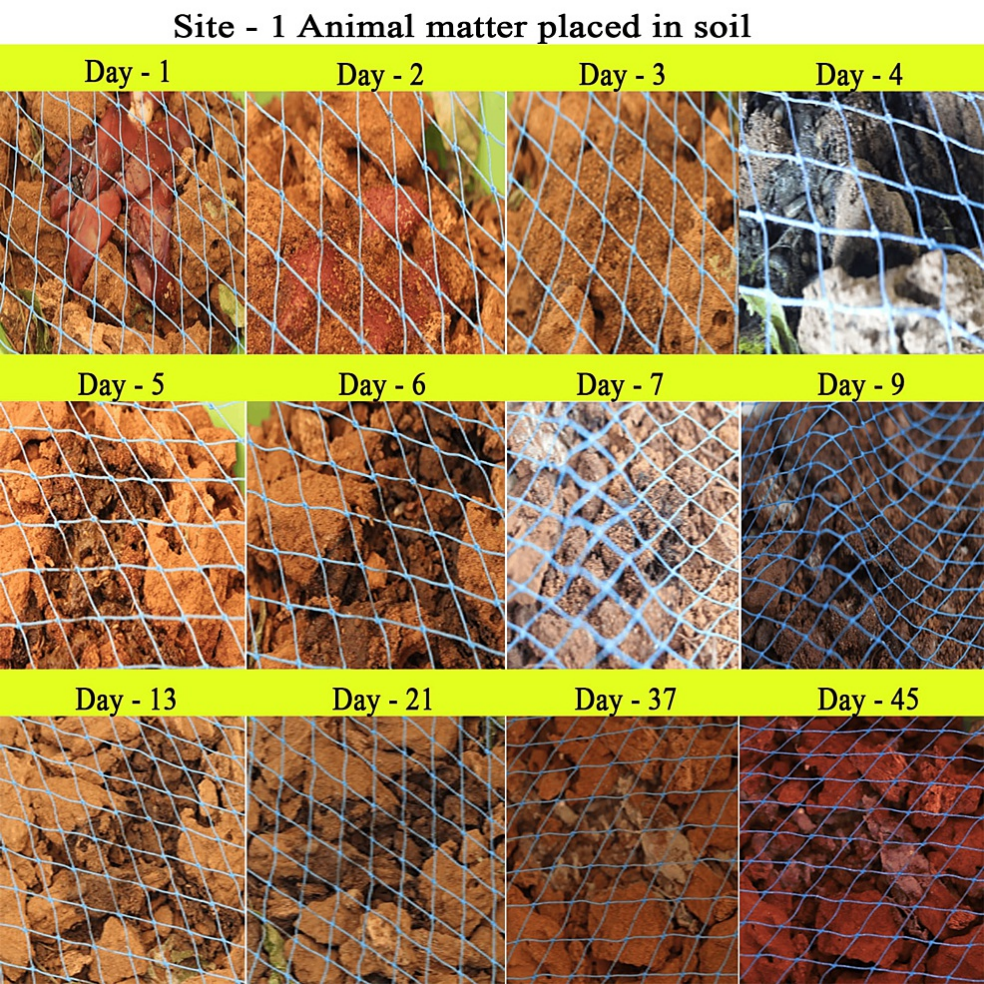
Animal Matter Placed in Soil

**Figure 4:**
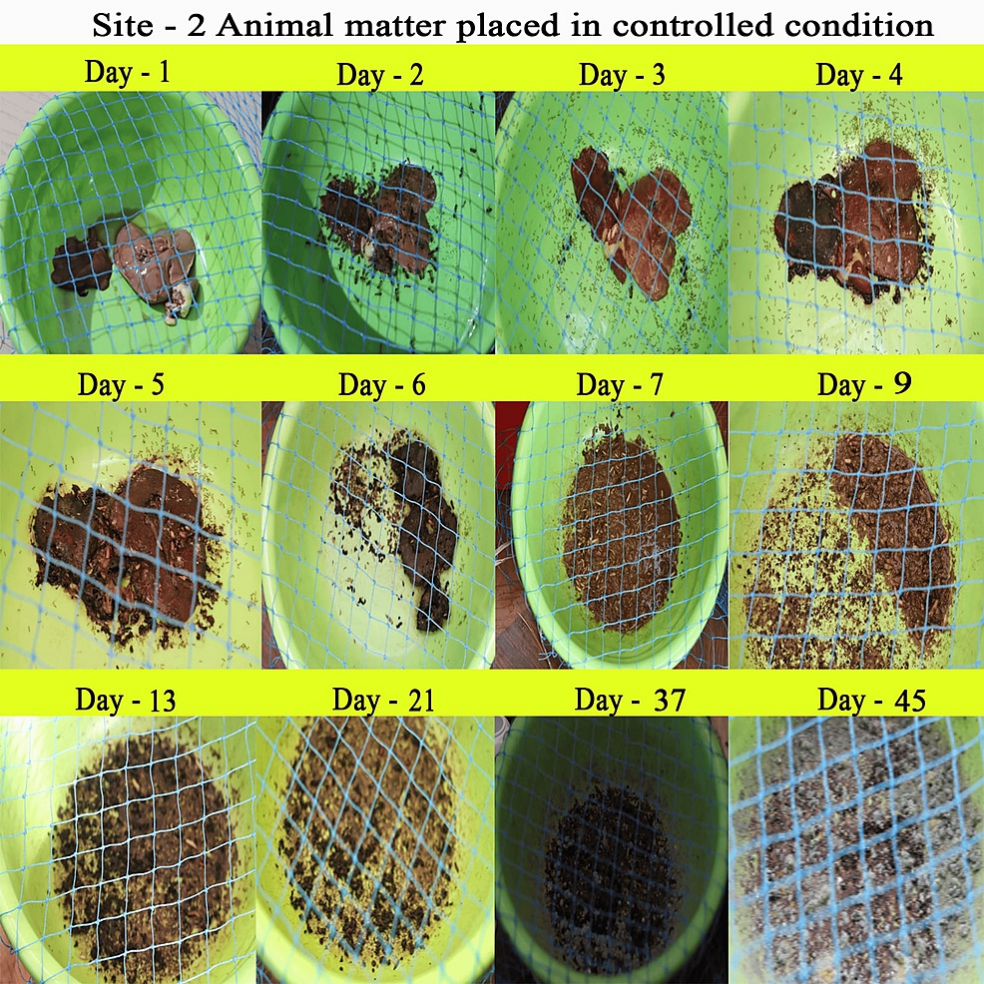
Animal Matter Placed in Controlled Condition

The complete decomposition of the decaying animal matter occurred within 35-40 days. We observed five decomposition phases, fresh, bloat, active decay, advanced decay, and dry stages, during the decomposition process (Figures 3, 4).

**Figure 5:**
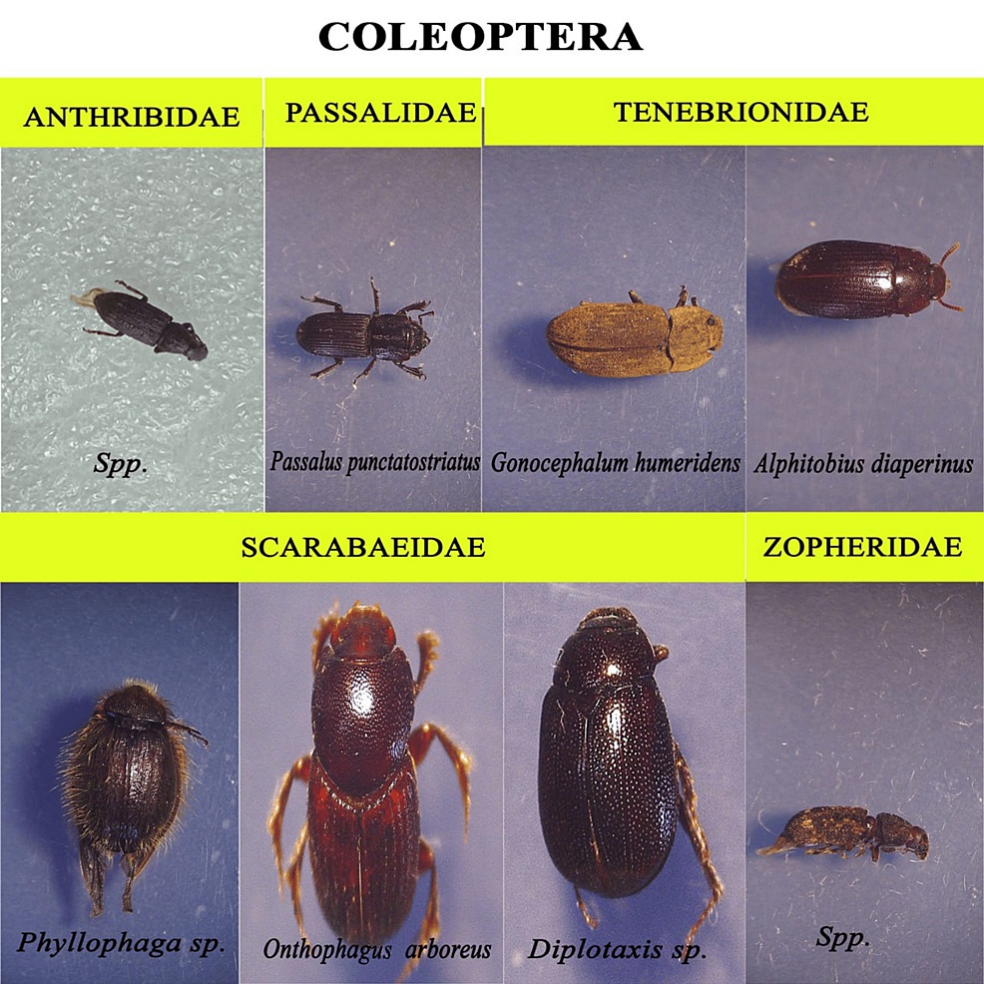
Coleoptera

**Figure 6:**
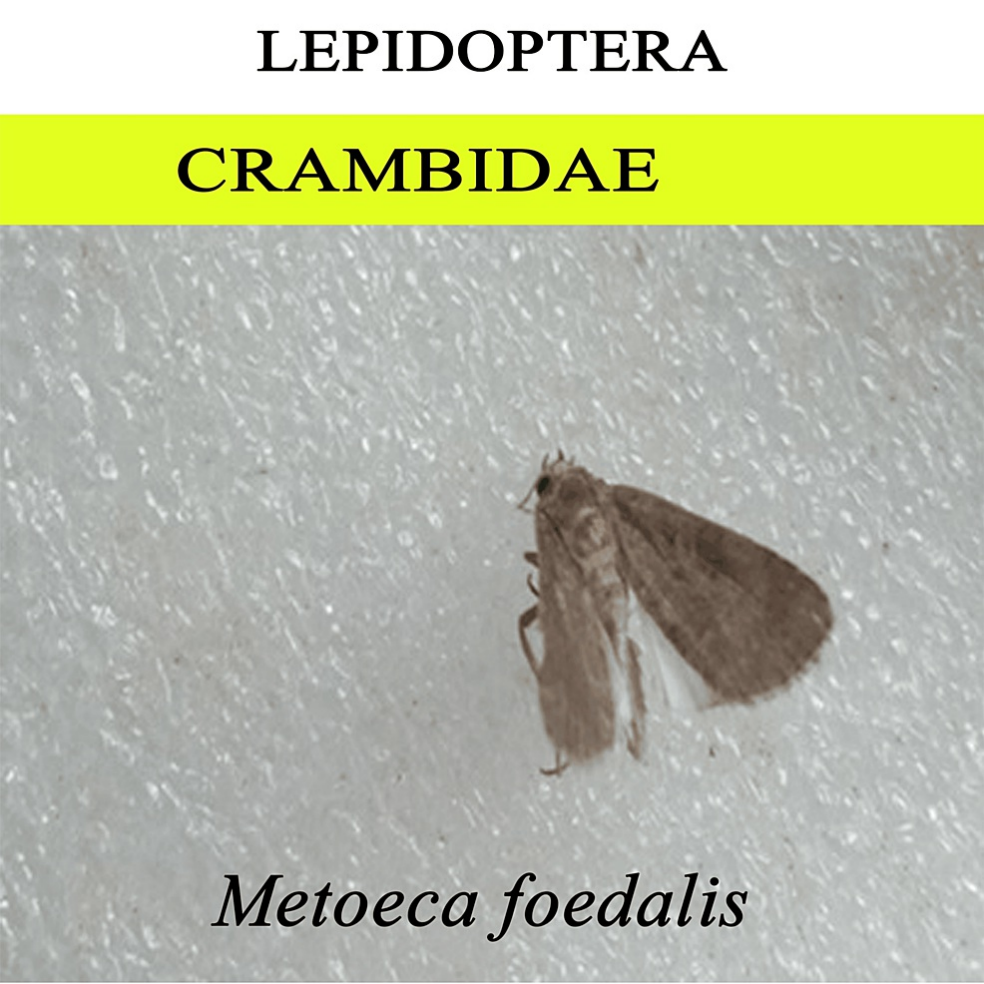
Lepidoptera

**Figure 7:**
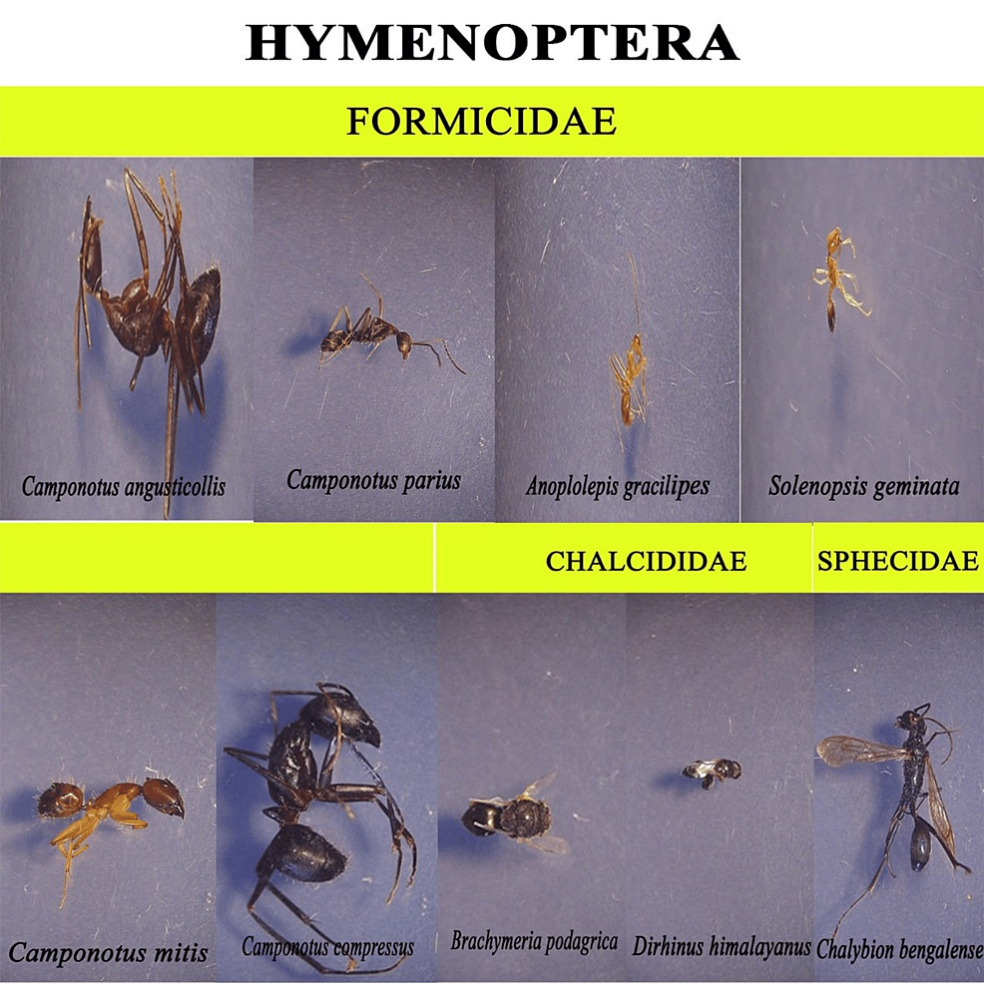
Hymenoptera

**Figure 8:**
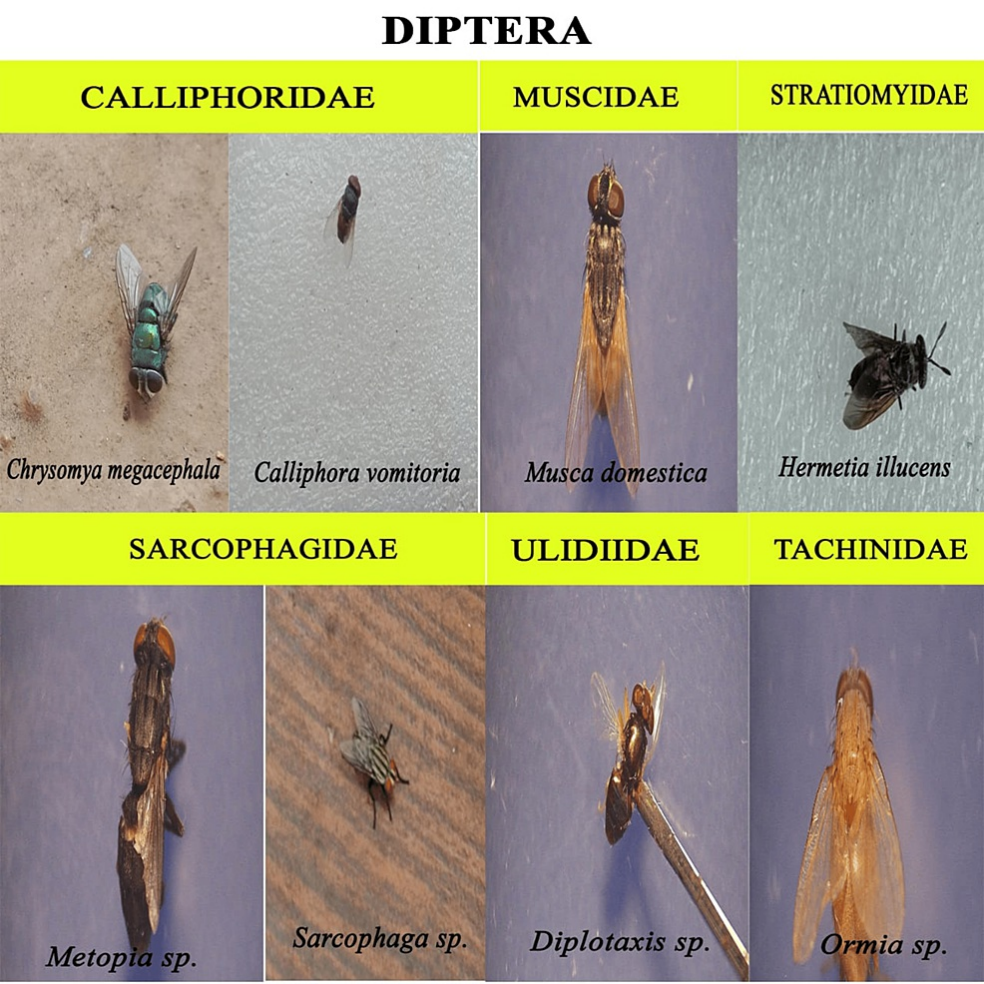
Diptera

**Figure 9:**
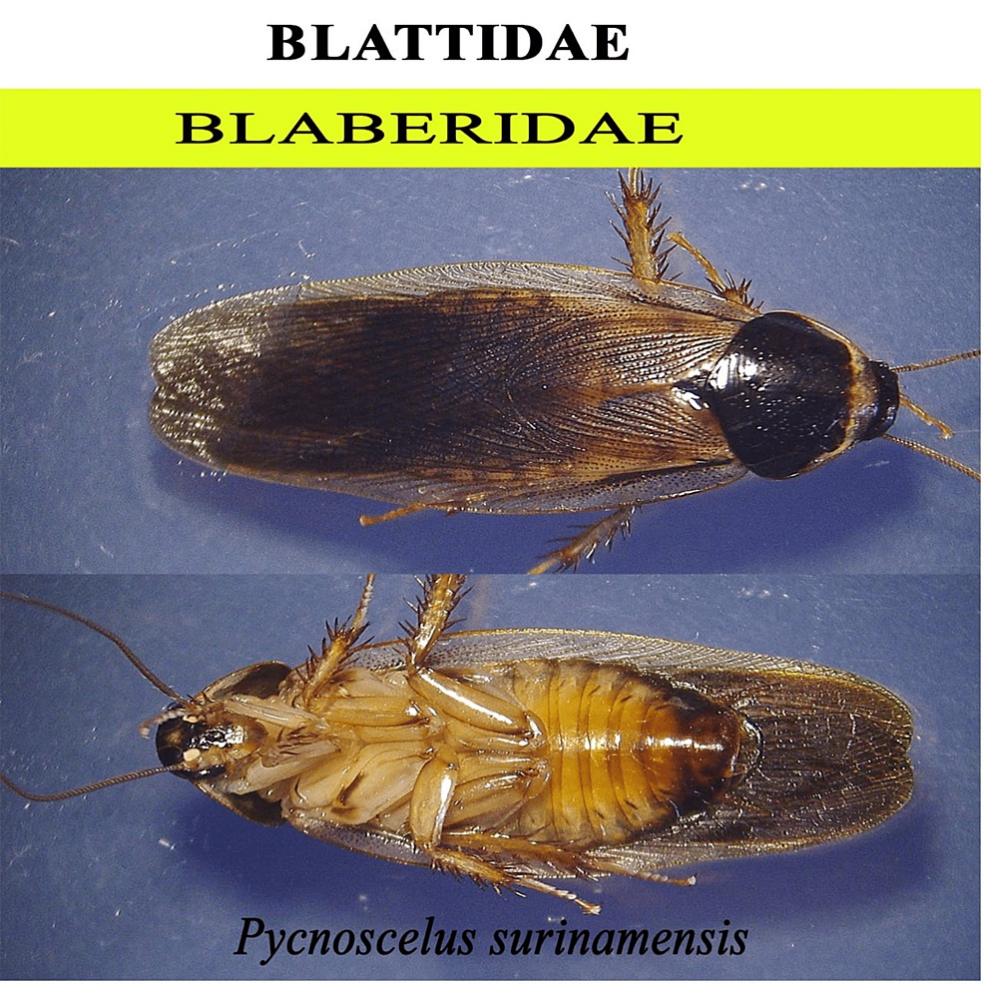
Blattidae

We recorded nearly 1500 insects from the decomposition of chicken liver, belonging to five orders (Coleoptera, Diptera, Hymenoptera, Blattidae, and Lepidoptera). Mites were the dominant arthropods found in the sample. However, the present study focused only on the entomofauna; therefore, the acarine decomposition is not taken to detailed study. For morphological identification, representative taxa from all the oriented groups were collected and recorded (Figures 5-9).

A brief description on the insect families observed

The members of Diptera and Coleoptera were the nominated members in the insects that visited the cadaver (Tables 1, 2).

**Table 1:** Diptera Associated With the Study Sample +, <5 specimens; ++, 5-50 specimens

Family	Genus	Species	Frequency
Calliphoridae	Chrysomya	megacephala	+
	Calliphora	vomitoria	+
Muscidae	Musca	domestica	++
Sarcophagidae	Metopia	sp.	++
	Sarcophaga	sp.	++
Stratiomyidae	Hermetia	illucens	++
Tachinidae	Ormia	sp.	++
Ulidiidae	Physiphora	sp.	+

**Table 2:** Coleoptera Associated With the Study Sample +, <5 specimens; ++, 5-50 specimens

Family	Genus	Species	Frequency
Anthribidae	Sp.	p.	+
Histeridae	Sp.	p.	++
Passalidae	Passalus	punctatostriatus	+
Scarabaeidae	Diplotaxis	sp.	++
	Onthophagus	arboreus	++
	Phyllophaga	sp.	+
Tenebrionidae	Alphitobius	diaperinus	+
	Gonocephalum	bilineatum	+
Zopheridae	Sp.	p.	++

Insect species identified are given in the tabular form with the frequency denoted as follows: +, <5 specimens; ++, 5-50 specimens; and +++, >50 specimens. The fly larvae are found to be responsible for the reduction and degradation of soft tissue. In the chain of succession, beetles generally arrive later on. They are mainly attracted to the desiccated tissue and are found to invade the decaying tissue once the flies are ready for pupation.

Entomo-succession

**Figure 10:**
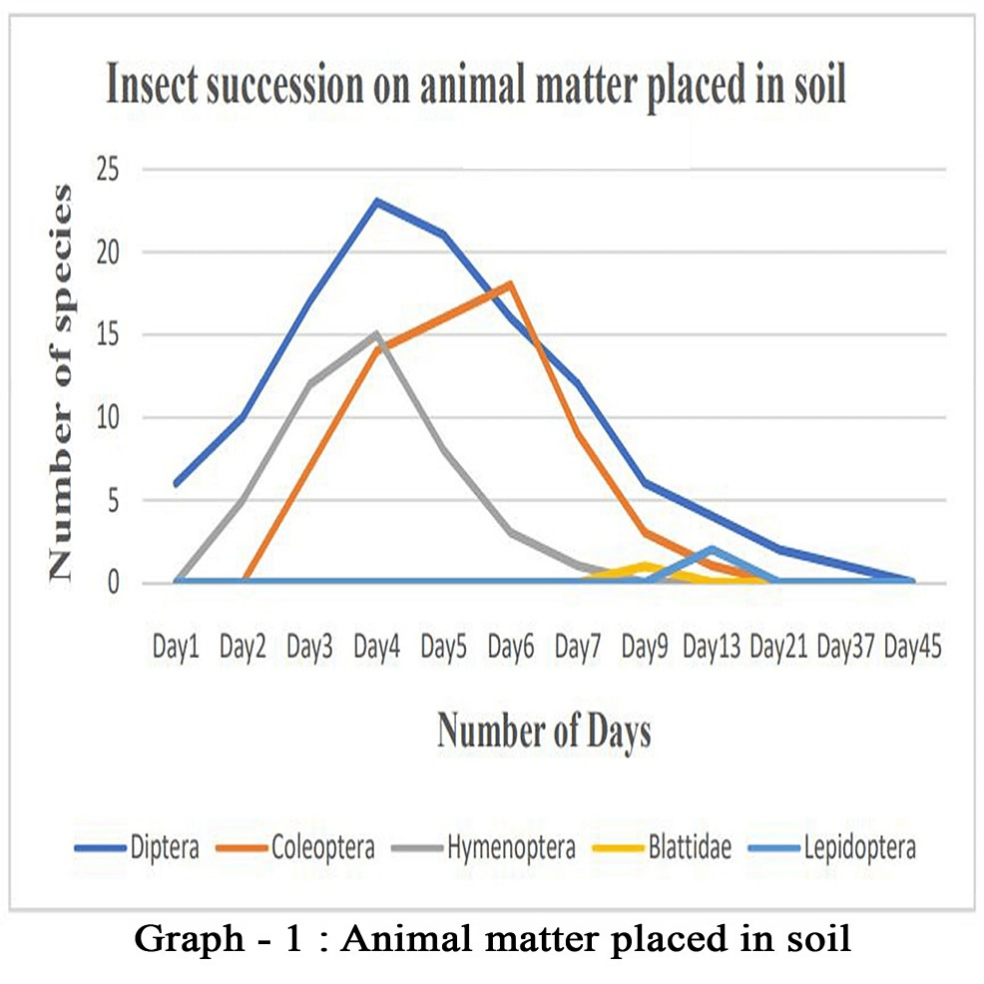
Entomo-Succession on Soil

**Figure 11:**
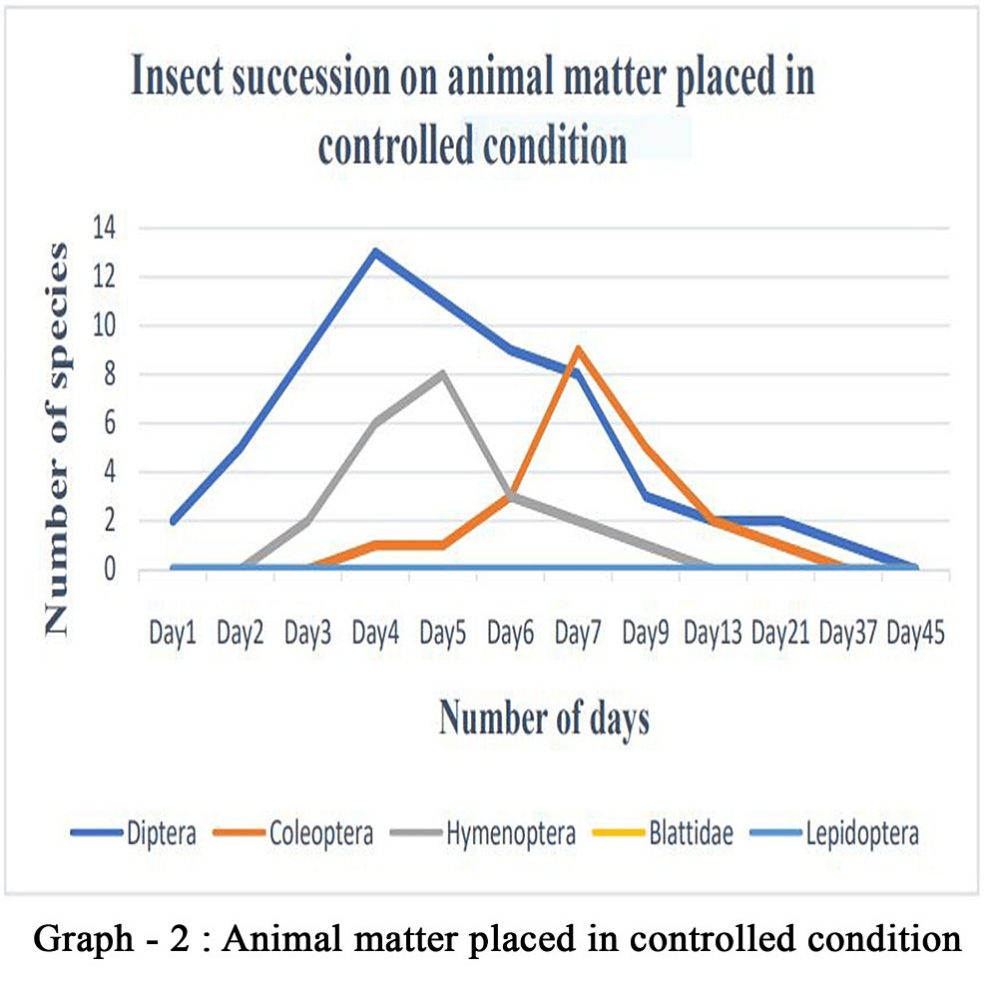
Entomo-Succession in Controlled Condition

For the graphical representation of entomo-succession on both control sample and the soil (Figures 10, 11), we used insects other than ants because ants are eusocial insects that can colonize the carrion very easily. Moreover, they feed on cadavers and act as decomposers. Therefore, ant faunas are immediate visitors on the cadaver irrespective of experimental setup, because they do not contribute substantial information on postmortem intervals. Ants are large in numbers (n > 100); it was omitted while preparing the graphical representation of succession. Insects have a shorter, simpler, and mappable lifespan; the stages of development can essentially be used as an important tool for estimating time since death.

## Discussion

In the present study, we focused on forensically important entomofauna on soft body tissue, by employing chicken liver. Although we found several insects, members of the orders Diptera, Coleoptera, and Hymenoptera were found to be important groups for forensic studies. There are several studies on these aspects; however, this study focuses on region-specific data on entomo-succession that help significantly with forensic investigation. The decomposition of soft body tissue includes five stages: fresh, bloat, active decay, advanced decay, and dry remains. The initial minutes of the experiment showed the presence of several insects, thus agreeing with the hypothesis that the degradation of the sample starts within an hour of death [17]. Nevertheless, carrion in the soil starts degrading much faster than in the control setup because of the active biota in the soil [18]. However, insects and the timing of their colonization will vary depending on the geographic region; therefore, this information may be used to detect whether or not carrion has been relocated [19]. Moreover, the rate of the decomposition of the carrion is influenced by the season, affecting the number and diversity of insects visiting the carrion. Our investigation was conducted in mid-April to mid-May under hot, humid weather conditions that are favorable for insects to colonize faster [20].

In this study, we documented 28 insect species from five orders and 17 families visiting the carrion. More than 1500 insects were observed during the study period, and around 100 were studied in detail. However, only 10 species from Diptera, Coleoptera, and Hymenoptera could be directly associated with the decomposition process, thus having forensic importance. We found that arthropod colonization follows a predictable sequence. Therefore, the presence of a particular group of organisms can easily be attributed to the temporal condition of the carcasses. In our study, at the fresh stage of decomposition, the insects first to arrive at the remains are Calliphoridae (*Chrysomya megacephala* and *Calliphora vomitoria*), known as blowflies. These arrived within minutes of exposure and, after scarring the tissue with their legs, deposited eggs within the hour. Adult flies of the family Sarcophagidae and Muscidae appeared next within 3-4 hours and also laid eggs in the tissue. Within a couple of days, small maggots were seen thriving in the bloated sample, and active feeding of the cadaver was observed. The common names for the members of the dipteran family Calliphoridae include blowflies, carrion flies, bluebottles, greenbottles, or cluster flies; they can detect the smell of carrion from two kilometers away and lay eggs on it. These blowflies were the first insects to touch a cadaver [21]. Moreover, their development on decaying matter is well studied. Therefore, blowflies are considered a tool for estimating the minimum postmortem interval (mPMI) for human corpses [22]. Importantly, blowflies can help determine whether the body was moved or the person used drugs prior to death, assuming colonization occurred after death [23].

In our experiment, ants and beetles were seen in higher numbers on the bloated stage. This was due to the activity of anaerobic bacteria [23]; gaseous changes occur in the decaying matter changing the color and structure of the substrate. During this stage, the odor of putrefaction was noticed, which attracted dipterans belonging to Sarcophagidae and Muscidae in large numbers. Members of the family Tachinidae were found during this stage, indicating that the host of the tachinid fly may have grown to the optimal stage of the parasitic fly. Ants were seen to feed on continuously on the animal matter, as well as on the eggs and young larvae of flies. We also found beetles of the family Histeridae during this phase, and they were often hidden below animal matter. Moreover, just prior to the competition of the stage, parasitic wasps such as *Brachymeria podagrica* and *Dirhinus himalayanus* both belonging to the family Chalcididae were found in both setups. These species are known to attack their dipteran hosts while they are in the pupal stage. From the incidence of these species in the samples, it could be determined that the immature flies feeding on the animal matter were in their pupation stage. These incidences can easily be correlated to the life history of the fly species, which can be used to calculate the time of death. The beginning of the third stage was marked with dipteran larvae feeding on the skin and the release of internal gases. Because of the liquefaction of tissues, the animal matter now appears wet. The larvae of Calliphoridae and Sarcophagidae are the dominant insect groups at this active decay stage. The number of Histeridae beetles increased, while the incidence and frequency of adult calliphorids and muscids decreased, marking a contrast in the succession of orders. Scarabaeid coleopterans were collected during this stage.

In the fourth stage, the advanced decay stage is marked by the removal of most of the flesh on the substrate. Strong odors of decomposition began to fade, and the last of all Diptera families, Stratiomyidae (*Hermetia illucens*), hovered over the remains. Some coleopterans also were seen opportunistically orienting to the remains. The final stage of the decomposition was the dry decay stage. There were very few remains at the end of the decomposition process. There was very little to no odor associated with the remains. Small bits of dried skin were only present at this stage, and very few organisms oriented toward the matter. Parasitic Hymenoptera other than the rest of the group of hymenopterans show the same importance in the study, their presence indicating a forensically important dipteran in its immature stage (pupa) thriving in the decaying animal matter. In the context of forensic analysis, it is thus important to discuss the life history, role, and importance of a dipteran species and associated parasitoids in the analysis of the death and decomposition of a carcass.

## Conclusions

We identified a variety of arthropod species involved in the breakdown of animal materials. The species belonging to 17 families in five orders, Coleoptera, Diptera, Hymenoptera, Blattidae, and Lepidoptera, were collected. The families of Diptera included Calliphoridae, Muscidae, Sarcophagidae, Stratiomyidae, Tachinidae, and Ulidiidae. The families of Coleoptera included Scarabaeidae, Tenebrionidae, Passalidae, Zopheridae, Anthribidae, and Histeridae. The families of Hymenoptera included Chalcididae, Formicidae, and Sphecidae. One species from the family Blaberidae (Blattidae) and one from the family Crambidae (Lepidoptera) were recorded from systematic taxonomic identification. After Acari, the two most important groups are Diptera and Coleoptera. The flies are early visitors to the remains because they are drawn to moist tissue. The loss of soft tissue is brought on by fly larvae. Beetles typically arrive later in the line of succession. The forensically important dipterans are in the families Calliphoridae, Sarcophagidae, and Muscidae, and other flies involved in the decomposition process are Tachinidae, Stratiomyidae, and Ulidiidae. The most important coleopteran families include Histeridae and Scarabaeidae. We also discovered two wasps associated with decomposition, *Brachymeria podagrica* and *Dirhinus himalayanus*, that can help estimate PMI in forensic analysis. However, further studies are required to model these organisms' life cycles for the most accurate PMI for forensic purposes.
